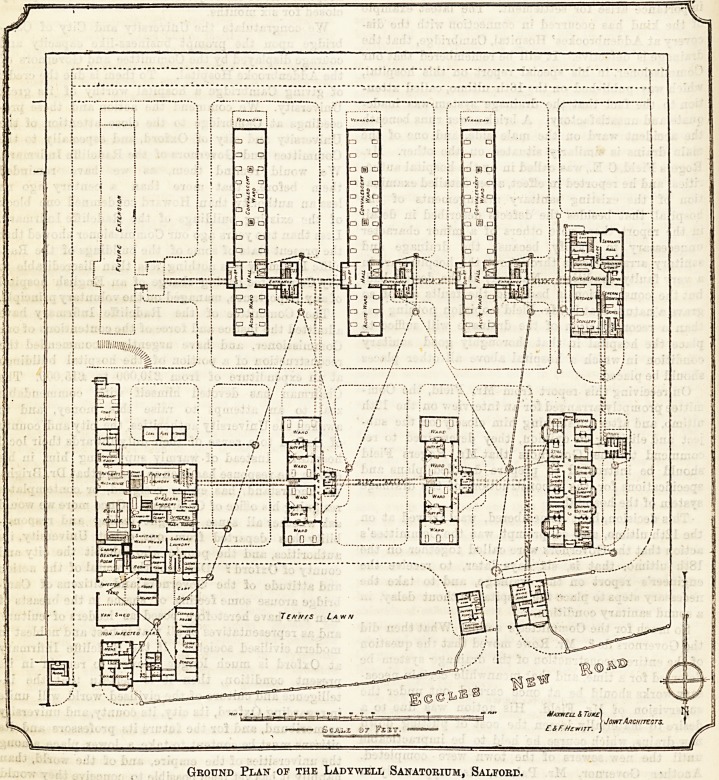# Ladywell Sanatorium, Salford

**Published:** 1892-08-20

**Authors:** 


### The Institutional Workshop.

### HOSPITAL CONSTRUCTION.

We have often called public attention to the marked difference which characterises the methods adopted by the hospital authorities of the Universities of Cambridge and Oxford when urgent questions of pressing importance arise for settlement. The latest example of the kind has occurred in connection with the discovery at Addenbrookes' Hospital, Cambridge, that the drainage is defective. It will be remembered that our Commissioner, in his special report on this hospital, which was published on the 16th ultimo, called attention to the fact that the drainage system was inadequate and unsatisfactory. A brick sewer runs beneath the accident ward on the male side, and one of the main drains is similarly situated on the other. Mr. Rogers Field, C E., was called in by the hospital authorities, and he reported in effect, after detailed examination of the existing sanitary arrangements of the hospital, that besides the defects described in detail in the report, there were others of a minor character unnecessary to specify, because the drainage and sanitary arrangements throughout the hospital are in a very faulty condition. Not only is the design bad, but the construction is bad, and the faults are of so grave a nature that in Mr. Field's opinion nothing less than a reconstruction of the drainage will suffice to place the hospital in that thoroughly good sanitary condition in which a hospital above all other places should be placed.

On receiving this report from Mr. Field, the Committee promptly arranged for an interview on the 12th ultimo, and after questioning him closely on the subject and eliciting his opinion, they determined to recommend to the Governors that Mr. Rogers Field should be instructed to prepare detailed plans and specifications for the reconstruction of the drainage system of the hospital.

This decision, be it remembered, was arrived at on the 12th ultimo, and so prompt was the Committee's action that the Governors were called together on the 18th ultimo, that is, six days later, to receive the engineer's report on the drainage, and to take the necessary steps to place the hospital, without delay, in a sound sanitary condition.

So much for the Committee's action. What then did the Governors do? Mr. Rowe moved that the question of the entire reconstruction of the drainage system be deferred for a time, and that meanwhile certain necessary works should be at once carried out under the supervision of Mr. Field. His action was due to a desire to materially lessen the cost of putting down new drains, which course he held to be impracticable until the new sewers of the town were completed. Another Governor, Mr. Palmer, also apparently in favour of delay, inquired how it was proposed to raise the large sum, amounting to thousands of pounds, rendered necessary if the hospital was to be redrained throughout? The answer was prompt and decisive. "That is an after-consideration," said the Chairman. In the result Mr. Rowe withdrew his proposal, and it was determined to adopt the Committee's report, and to forthwith proceed with the redrainage of the hospital, Mr. Palmer being the only Governor who voted against this course. The Drainage Committee was reappointed with power to consider what arrangements could be made for the accommodation of the patients during the execution of the work. It appears that the hospital will be entirely closed for six weeks, and partly closed for six months.

We congratulate the University and City of Cambridge upon the prompt business-like capacity and courage displayed by the Committee and Governors of the Addenbrooke Hospital. To them is due the credit of giving Cambridge a hospital worthy of its great University. We commend the action and these proceedings at Cambridge to the close attention of the University and city of Oxford, and especially to the Committee and Governors of the Radcliffe Infirmary. We would remind them, as we have reminded them before, that more than a century ago no less an authority than Howard condemned one block of the existing buildings of the Radcliffe Infirmary. Less than two years ago our Commissioner showed that the present state of some of the buildings of the Radcliffe Infirmary was nothing less than discreditable to any Committee having charge of an English hospital of any importance, managed on the voluntary principle.

The Committee of the Radcliffe Infirmary have admitted the justice and force of the contentions of our Commissioner, and have urgently recommended the reconstruction of a portion of the hospital buildings at an expenditure of from £10,000 to £15,000. The Chairman has devoted himself with commendable zeal to an attempt to raise this money, and to awaken the University authorities, the city and county of Oxford to a sense of their duty towards their local hospital. Instead of warmly supporting him in his efforts, the response has been so meagre that Dr. Bright, we understand, has either resigned, or contemplates resigning his office of Chairman. Once more we would ask whether all sense of right feeling and responsibility has departed from our oldest University, its authorities, and the people who inhabit the city and county of Oxford? Does not the recital of the action and attitude of the Governors and citizens of Cambridge arouse some feeling of shame in the breasts of men who have heretofore posed as leaders of culture, and as representatives of all that is best and noblest in modern civilised society? If the Radcliffe Infirmary at Oxford is much longer allowed to remain in its present condition, then it is certain that the intelligence and culture of the civilised world will unite in regarding Oxford, its city, its county, and university as moribund, and for the future its professors and its citizens must be content to take a lower place among the universities of the empire, and of the world, than happily it has yet been possible to conceive they would be content to occupy.

---

### THE LADYWELL SANATORIUM, SALFORD.

The completion of a new and well-equipped Hospital for Infectious Diseases for the County Borough of Salford is an event on which the Health Officers are greatly to be congratulated. The old fever hospital in Cross Lane known as the Wilton Hospital was a row of five ordinary dwelling houses converted by various alterations and additions into what was at best but an imperfect substitute for an efficient hospital. Dr. Thorne Thorne refers to it in his report on Infectious hospitals as a "conspicuous illustration" of a hospital provided under the influence of panic, "in a very imperfect manner, and at a needlessly large cost."

The hospital just completed wipes away the reproach attaching to the Sanitary Authorities of Salford, and reflects great credit on all concerned, not the least of whom is Dr. Tatham, the Medical Officer of Health for Manchester, who was for many years the Health Officer for Salford.

The site, actually belonging to the Corporation, is about 13 acres in extent, but at present only about 7½ acres are used for hospital purposes.

The main entrance to the hospital is at the north-west corner of the site. On the right of this is a porter's lodge, which contains accommodation for a married porter, as well as rooms for unmarried men employed about the hospital.

To the left is a one-storey building containing the discharge rooms for patients, with a waiting-room for visitors. The discharge rooms consist of an undressing room, bathroom, and dressing-room. The patient on his discharge undresses in the first room, takes his bath, and dresses in his own disinfected clothes in the third room, and so passes out of the hospital grounds. The waiting-room serves the double purpose of an inquiry-room for patients' friends, and of a room where parents or friends can receive patients on their discharge.

The ward pavilions are five in number. Two of these are intended as isolation blocks for the reception of doubtful cases, cases requiring separation for any reason, and for use when only a few cases are under treatment at one time; they

Ground Plan of the Ladywell Sanatorium, Salford.

are two storeys in height, and contain on each floor two wards for three beds, and two for two beds each. These wards are arranged in pairs, a ward scullery being interposed between each large and small ward; and all the rooms are entered from an open verandah, off which the closets are projected.

The three ward pavilions are also two storeys in height. On each floor is an acute ward for six beds and a convalescent ward for eighteen beds, and between the two wards is a large ward scullery, the enclosing wall of which is a glazed partition. Opposite the ward scullery is a bath-room and a linen closet. The closets and staircase to the upper floor are placed in a wing separated from the main building by a lobby provided with casement windows on each side, which are intended to be used in winter only, the sides being left open during the summer. Within the well of the staircase is a lift for conveying patients to the floor above. At the south end of the pavilion it is intended at some future time to erect covered balconies or "sun-rooms." The acute wards are provided with large, open fire-places, with mantel-pieces of faience ware; and the convalescent wards have each two of Musgrave's ventilating stoves. In addition to these all the wards have steam coils under every alternate window, fitted with apparatus for admitting fresh air from the outside. Both gas and electric lighting are provided, and the gas lights have ventilating tubes to carry off the products of combustion. The windows are made to open without break from floor to ceiling—an excellent arrangement for obtaining a thorough flush of air, but one which will have to be used with caution on the upper floor.

The administration offices are divided among three buildings: (1) The quarters for resident officers and servants; (2) the kitchen and stores; and (3) the laundry and disinfection department.

The officers' block, which is placed to the west of the isolation blocks, contains the committee-room, rooms for the resident medical officer and dispenser, the nurses' home and lady superintendent's rooms, and bed-rooms for servants. The nursing home is provided with a separate entrance and staircase, and affords accommodation for thirty-three nurses, each having a separate bed-room. There are sitting-rooms for sisters and nurses, and two waiting-rooms for visitors.

The kitchen block is placed to the west of the ward pavilions, and is two storeys high. Besides the kitchen offices there are extensive stores, sewing-rooms, and dispensary.

The laundry and disinfection department is contained in an extensive range of buildings to the north-east of the site. There are here two complete laundries—one for the patients, the other for the officers. The "sanitary department" comprises a disinfecting-room, fitted with a "Lyons" steam apparatus; a carpet-beating room, with a machine supplied by Messrs. Thomas and Taylor; a destructor for burning the hospital refuse, erected by Messrs. Manlove, Alliott, and Co.; and rooms for washing infected clothing sent in from outside. This latter has not yet been fitted up, but the idea is an excellent one, and, so far as we know, is one that has never yet been adopted elsewhere.

Close to the laundries is the boiler and engine house. Here also are placed the dynamos for working the electric light. The mortuary and post-mortem room also form part of this group of buildings.

Close to the north-east entrance are the following: A waiting-room for mourners; a small lodge contains rooms for the men employed in this part of the hospital; an ambulance house, and a range of stabling.

The drainage appears to be devised on a simple and efficient system, and ample provision is made for flushing.

The whole of the steam pipes, gas and water pipes, electric lighting mains, bell wires and telephone wires, are carried in subways which connect the various blocks.

The general planning and careful attention to detail reflect very great credit upon the joint architects, Messrs. Maxwell and Tuke and Messrs. E. and F. Hewitt, both of Manchester.

## Figures and Tables

**Figure f1:**